# Unlocking the Potential of Immunotherapy in Cardiovascular Disease: A Comprehensive Review of Applications and Future Directions

**DOI:** 10.7759/cureus.42790

**Published:** 2023-08-01

**Authors:** FNU Jyotsna, Jibran Ikram, FNU Nageeta, FNU Komal, FNU Anjlee, Harshkumar Patel, Taleb Nassri, Meena Kumari, Rajesh Kumar, Syeda Urooba Shah, Maham Kashif, Giustino Varrassi, Satesh Kumar, Tirath Patel

**Affiliations:** 1 Medicine, Dr. B.R. Ambedkar Medical College & Hospital, Mohali, IND; 2 Orthopaedics and Trauma, Rehman Medical Institute, Peshawar, PAK; 3 Medicine, Ghulam Muhammad Mahar Medical College, Sukkur, PAK; 4 Medicine, Chandka Medical College, Larkana, PAK; 5 Internal Medicine, PDU (Pandit Dindayal Upadhyay) Medical College, Rajkot, IND; 6 Medicine, Heart and Vascular Institute, Dearborn, USA; 7 Internal Medicine, Dow University of Health Sciences, Karachi, PAK; 8 Business Intelligence and Data Analytics, Westcliff University, Irvine, USA; 9 Medicine, Jinnah Sindh Medical University, Karachi, PAK; 10 Medicine, Khawaja Muhammad Safdar Medical College, Wazirabad, PAK; 11 Pain Medicine, Paolo Procacci Foundation, Rome, ITA; 12 Medicine and Surgery, Shaheed Mohtarma Benazir Bhutto Medical College, Karachi, PAK; 13 Medicine, American University of Antigua, St. John, ATG

**Keywords:** applications, cardiac risk factors and prevention, immune, therapeutic, cardiovascular, immunotherapy

## Abstract

Immunotherapy has emerged as a pioneering therapeutic approach that harnesses the immune system's abilities to combat diseases, particularly in the field of oncology where it has led to significant advancements. However, despite its significant impact in the field of oncology, the potential of immunotherapy in the context of cardiovascular disease (CVD) has not been thoroughly investigated. The purpose of this narrative review is to address the existing knowledge and potential uses of immunotherapy in the field of cardiovascular disease (CVD), with the intention of filling the existing gap in understanding. Furthermore, the review thoroughly examines the future prospects of this swiftly advancing field, providing insights into the aspects that necessitate further investigation and addressing the forthcoming challenges. The review is organized into four distinct sections to enhance comprehension. The first section introduces immunotherapy, presenting the fundamental concepts and principles. The second section explores the immunomodulatory mechanisms in cardiovascular disease (CVD), with a specific focus on the intricate interplay between the immune system and the development of cardiovascular pathogenesis. The utilization of immunotherapy in specific cardiovascular conditions will be examined, investigating the application of immunotherapy in the context of different cardiovascular diseases. The future prospects and challenges in immunotherapy for cardiovascular diseases will be discussed, highlighting the potential areas for future research and addressing the barriers that must be overcome to effectively implement immunotherapeutic interventions in the management of cardiovascular diseases.

## Introduction and background

Immunotherapy has significantly transformed the field of medicine by introducing a fundamental change in our approach to disease treatment. Immunotherapy, in contrast to conventional therapies that directly focus on the disease, utilizes the inherent capabilities of the immune system to effectively combat various diseases. The process entails the activation or modulation of the immune response to effectively identify and eliminate cells or tissues affected by the disease. The implementation of this innovative approach has significantly reshaped the medical treatment landscape, presenting novel opportunities for the management of diverse diseases.

Immunotherapeutic approaches encompass a diverse array of strategies that are specifically formulated to stimulate or modulate the immune system's response against various diseases. These approaches can be classified into various modalities, each characterized by its distinct mechanism of action. One notable strategy involves the utilization of immune checkpoint inhibitors, which function by obstructing the inhibitory signals that tumor cells exploit in order to evade immune recognition and elimination. The use of antibodies that target immune checkpoints, such as programmed cell death protein 1 (PD-1)/PD-1 ligand (PD-L1) and cytotoxic T-lymphocyte-associated antigen 4 (CTLA-4), has demonstrated significant efficacy in augmenting the immune response against malignant cells [1]. Adoptive cell therapies are considered a highly effective form of immunotherapy. This process entails the introduction of genetically modified immune cells, such as chimeric antigen receptor (CAR) T cells, which have been engineered to express distinct receptors capable of identifying and selectively attacking cancer cells. The application of CAR T cell therapy has exhibited noteworthy clinical results in specific hematologic malignancies [2]. Therapeutic vaccines have the objective of activating the immune system to identify and initiate a focused immune response against particular antigens present in cancer cells. These vaccines have the potential to augment the immune response and facilitate the targeted elimination of cancer cells [3]. Cytokine-based therapies encompass the administration of cytokines, such as interleukins or interferons, with the aim of regulating the activity of the immune system. These therapeutic interventions have the potential to augment immune responses and facilitate antitumor effects [4].

Although immunotherapy has demonstrated significant success in the field of oncology, its potential applications in cardiovascular disease (CVD) have received limited exploration thus far. Nevertheless, recent research has provided valuable insights into potential targets and the immunomodulatory mechanisms associated with cardiovascular disease (CVD). Inflammatory processes are of utmost importance in the development of cardiovascular diseases (CVD), encompassing conditions such as atherosclerosis, myocardial infarction, and heart failure. The dysregulation of immune cells, specifically macrophages, T cells, and dendritic cells, is a significant factor in the development of chronic inflammation and the progression of cardiovascular disease [5]. Hence, the strategic targeting of these immune cells and the modulation of the inflammatory response via immunotherapy offer a promising approach for therapeutic intervention in cardiovascular disease (CVD). In addition, recent findings indicate that immune checkpoint molecules, namely PD-1/PD-L1 and CTLA-4, which have been extensively utilized in cancer immunotherapy, are also present in the cardiovascular system and could potentially impact the progression of cardiovascular disease [6]. The manipulation of immune checkpoint pathways within the context of cardiovascular disease (CVD) has the potential to effectively regulate the immune response and mitigate the progression of the disease.

In summary, immunotherapy has emerged as a transformative approach in the field of medicine, utilizing the inherent capabilities of the immune system to effectively combat various diseases. The success achieved in the field of oncology has opened up opportunities for further exploration of its applications in various other domains, such as cardiovascular disease. Immunotherapeutic interventions have the potential to provide innovative strategies for managing and treating cardiovascular disease (CVD) by specifically targeting immune cells, modulating the inflammatory response, and exploring immune checkpoint pathways.

## Review

Immunomodulatory mechanisms in CVD

The Role of Inflammation in the Pathogenesis of Cardiovascular Disease

Inflammation plays a crucial role in the development of cardiovascular disease (CVD). Chronic inflammation within the arterial wall, especially in the context of atherosclerosis, plays a significant role in driving the progression of the disease [7]. The inflammatory response entails the activation of immune cells and the secretion of pro-inflammatory cytokines and chemokines, resulting in the recruitment and accumulation of immune cells at the site of injury [8]. Atherosclerosis, the primary underlying factor contributing to most cardiovascular diseases (CVDs), is distinguished by the development of plaques within the walls of arteries. Endothelial dysfunction, which can be triggered by risk factors such as elevated cholesterol levels or hypertension, results in the infiltration of immune cells, particularly monocytes and macrophages, into the arterial wall. The immune cells effectively phagocytose altered lipoproteins, specifically oxidized low-density lipoproteins (LDL), resulting in their conversion into foam cells. The presence of foam cells plays a significant role in the development of fatty streaks, which are a characteristic feature of early-stage atherosclerosis [9]. The immune response elicited by these foam cells laden with lipids initiates an inflammatory cascade. Inflammatory cytokines, such as interleukin-1 (IL-1), interleukin-6 (IL-6), and tumor necrosis factor-alpha (TNF-alpha), are secreted, facilitating the recruitment of supplementary immune cells, including T cells and dendritic cells. T cells, specifically, engage in interactions with endothelial cells, thereby enhancing the inflammatory response via the secretion of cytokines and chemokines. This process contributes to endothelial dysfunction and promotes the progression of plaque formation [10].

Immune Cell Subsets and Their Role in Cardiovascular Disease (CVD)

Various subsets of immune cells play distinct roles in the pathogenesis of cardiovascular disease (CVD). Macrophages, as previously stated, have a pivotal role in the development of atherosclerosis. Foam cells are formed when macrophages engulf oxidized LDL, thereby playing a significant role in the development of plaque [11]. Macrophages are known to secrete pro-inflammatory cytokines, matrix metalloproteinases (MMPs), and various enzymes that contribute to the process of inflammation and destabilization of plaque [12]. T cells play a significant role in cardiovascular disease (CVD). CD4+ T cells, commonly known as helper T cells, possess the ability to undergo differentiation into distinct subsets that exhibit specialized functions. T-helper 1 (Th1) cells are responsible for the production of pro-inflammatory cytokines, including interferon-gamma (IFN-gamma). These cytokines play a crucial role in promoting inflammation and contributing to the destabilization of plaque [13]. T-helper 2 (Th2) cells, conversely, secrete anti-inflammatory cytokines, including IL-4 and IL-10, which potentially play a protective role in restricting the advancement of plaque. T-helper 17 (Th17) cells play a significant role in the perpetuation of chronic inflammation and have been linked to heightened vulnerability to plaque formation [14]. Regulatory T cells, also known as Tregs, fulfill a crucial regulatory function within the immune response and possess the capability to suppress inflammation and promote plaque stabilization [15]. In addition to other immune cell subsets, such as natural killer (NK) cells and B cells, there is also a contribution to the inflammatory environment in cardiovascular disease (CVD). Natural killer (NK) cells have been associated with the advancement and destabilization of plaque, primarily due to their ability to produce pro-inflammatory cytokines and exhibit cytotoxic activity [16]. In contrast, B cells have the ability to generate autoantibodies that play a role in the progression of atherosclerosis and systemic inflammation [17].

Immunomodulation as a Therapeutic Approach in Cardiovascular Disease (CVD)

Considering the significant impact of immune cell activation and inflammation on cardiovascular disease (CVD), the concept of immunomodulation has arisen as a promising therapeutic approach. The objective is to regulate the immune response, reduce inflammation, and enhance plaque stability. Numerous methodologies have been explored in order to attain immunomodulation in cardiovascular disease (CVD). One potential strategy involves the precise targeting of distinct subsets of immune cells or modulating their functions. For instance, therapeutic interventions targeting the reduction of macrophage infiltration or the inhibition of pro-inflammatory cytokines they generate have exhibited promising results in preclinical and initial clinical investigations. Investigations are currently underway to explore strategies that specifically target T cells, including the modulation of Th1 and Th2 responses or the promotion of Treg activity.

An alternative strategy entails the utilization of immune checkpoint inhibitors, which effectively obstruct inhibitory molecules and augment immune responses. The regulation of T-cell activation and function is mediated by immune checkpoints, including PD-1/PD-L1 and CTLA-4 [18]. By obstructing these checkpoints, it is possible to augment immune responses against plaque components, which could potentially result in the regression or stabilization of plaque. In addition, there is ongoing research into the development of vaccines that target specific antigens found in atherosclerotic plaques. This approach is being explored as a potential strategy to stimulate an immune response against the components of the plaques. The objective of these vaccines is to stimulate an anti-inflammatory or regulatory immune response in order to modulate the progression of plaque.

Despite the potential of immunomodulatory strategies, there are various challenges that need to be addressed. It is crucial to achieve accurate targeting of immune cells or pathways while ensuring the integrity of the overall immune response. Furthermore, the presence of potential off-target effects and the necessity to carefully manage immune activation and suppression introduce further intricacies. Additional research is necessary to enhance our comprehension of the immunomodulatory mechanisms in cardiovascular disease (CVD) and to establish therapeutic interventions that are both effective and safe.

In conclusion, immunomodulatory mechanisms have a significant impact on the development of cardiovascular disease (CVD), with inflammation serving as a primary catalyst. Various subsets of immune cells, such as macrophages, T cells, and dendritic cells, play a role in the development and progression of plaque. The utilization of immunomodulation as a therapeutic approach in cardiovascular disease (CVD) aims to mitigate inflammation, stabilize plaques, and diminish the occurrence of cardiovascular events. Ongoing research is focused on the targeting of specific subsets of immune cells, the modulation of immune responses, and the exploration of immune checkpoint pathways. However, additional research is required to optimize these methodologies and address the challenges associated with their efficient and secure implementation in the management of cardiovascular disease (CVD).

Applications of immunotherapy in specific cardiovascular conditions

The Relationship Between Atherosclerosis and Immune Checkpoint Inhibitors

Atherosclerosis is a persistent inflammatory condition characterized by the development of atherosclerotic plaques within the arterial wall, thereby contributing to the onset of cardiovascular disease [19]. The utilization of immunotherapy, particularly immune checkpoint inhibitors, has demonstrated promise in the modulation of the immune response and the reduction of plaque burden in atherosclerosis [20]. Extensive research has been conducted on the use of immune checkpoint inhibitors, specifically antibodies that target programmed cell death protein 1 (PD-1) and its ligand PD-L1, in the field of cancer immunotherapy. These inhibitors interfere with the inhibitory signals that tumors utilize to evade immune surveillance [21]. In the context of atherosclerosis, previous studies have shown that inhibiting the PD-1/PD-L1 pathway augments the immune response toward atherosclerotic plaques. As a result, there is a reduction in plaque size, a decrease in lipid accumulation, and an improvement in plaque stability [22]. In addition, research conducted on mice that lack PD-L1 has demonstrated a heightened vulnerability to atherosclerosis. This underscores the significance of immune checkpoints in the regulation of plaque advancement [23]. The current stage of clinical trials exploring the application of immune checkpoint inhibitors in the treatment of atherosclerosis is in its preliminary phase. Nevertheless, preliminary findings suggest that immune checkpoint inhibitors possess the capacity to significantly diminish plaque accumulation and stabilize susceptible plaques among individuals diagnosed with atherosclerosis [24].

Myocardial Infarction and Cellular Immunotherapies

Myocardial infarction, also referred to as a heart attack, is a medical condition characterized by the obstruction of blood flow to the heart muscle, leading to the demise of cardiac cells. The immune response that occurs after myocardial infarction is of utmost importance in the process of tissue repair and healing [25]. Immunotherapeutic strategies that specifically target cellular components have demonstrated promising results in facilitating the regeneration of cardiac tissue and enhancing outcomes following myocardial infarction [26]. Research has been conducted on cellular immunotherapies, specifically the utilization of mesenchymal stem cells (MSCs) and cardiac progenitor cells (CPCs), as prospective therapeutic interventions for myocardial infarction [24]. Mesenchymal stem cells (MSCs) exhibit immunomodulatory characteristics and have the ability to regulate the inflammatory response, mitigate scar formation, and facilitate tissue regeneration. Preclinical studies have provided evidence that the transplantation of mesenchymal stem cells (MSCs) following myocardial infarction can potentially enhance cardiac function, decrease the size of the infarcted region, and promote angiogenesis [27].

Contrary to that, cardiac progenitor cells (CPCs) possess the capability to differentiate into diverse types of cardiac cells and actively participate in the process of tissue repair. The transplantation of cardiac progenitor cells (CPCs) subsequent to myocardial infarction has exhibited promising results in enhancing cardiac function and mitigating adverse remodeling [26]. Cellular immunotherapies for myocardial infarction are currently in the experimental phase; however, preliminary clinical trials have demonstrated the safety and viability of these strategies. Additional research is required to enhance the optimization of cell types, delivery methods, and intervention timing in order to maximize the therapeutic efficacy of myocardial infarction treatment.

Heart Failure and Immunotherapies Utilizing Cytokines

Heart failure is a multifaceted syndrome characterized by compromised cardiac function and is commonly linked to chronic inflammation and immune dysregulation. Cytokine-based immunotherapies have emerged as potential therapeutic strategies for heart failure. These therapies aim to modulate the immune response by administering specific cytokines [28]. Interleukins, specifically interleukin-1 (IL-1) and interleukin-6 (IL-6), play a crucial role as mediators of the inflammatory response in cases of heart failure. Increased levels of interleukin-1 (IL-1) and interleukin-6 (IL-6) have been linked to detrimental cardiac remodeling and unfavorable outcomes in individuals with heart failure [29]. The targeting of these cytokines through the use of specific inhibitors or neutralizing antibodies has demonstrated promising results in preclinical studies. These approaches have been effective in reducing inflammation, enhancing cardiac function, and mitigating adverse remodeling [30].

In addition, immune modulatory cytokines, specifically interleukin-10 (IL-10) and transforming growth factor-beta (TGF-beta), have demonstrated promising capabilities in facilitating cardiac repair and regeneration [31]. These cytokines have the ability to inhibit the inflammatory response, promote tissue repair mechanisms, and induce angiogenesis. Preclinical studies have provided evidence of the advantageous effects of interleukin-10 (IL-10) and transforming growth factor-beta (TGF-beta) in enhancing cardiac function and diminishing fibrosis in experimental models of heart failure [32]. While cytokine-based immunotherapies for heart failure are currently in the preliminary stages of research, they exhibit potential as viable treatments for modulating the immune response, mitigating inflammation, and facilitating cardiac repair in individuals diagnosed with heart failure.

Transplant-Associated Vasculopathy and Immune Tolerance Induction

Transplant-associated vasculopathy (TAV) is a frequently observed complication that arises subsequent to the transplantation of solid organs. The condition is distinguished by the emergence of vascular lesions within the vasculature of the transplanted organ [23]. The immune response directed toward the transplanted organ is of utmost importance in the progression of transplant-associated vasculopathy (TAV). Immunotherapeutic strategies targeting the induction of immune tolerance have demonstrated potential in the prevention or treatment of TAV. An effective strategy for inducing immune tolerance involves the utilization of regulatory T cells (Tregs). Regulatory T cells (Tregs) are a distinct subgroup of T cells that possess immunosuppressive characteristics, enabling them to effectively suppress the activation and functionality of effector T cells [20]. Preclinical studies have provided evidence that the adoptive transfer of regulatory T cells (Tregs) can effectively inhibit the immune response directed toward the transplanted organ and mitigate the occurrence of transplant-associated vasculopathy (TAV) [14].

In addition to regulatory T cells (Tregs), alternative approaches to promote immune tolerance encompass the utilization of immune modulatory agents, such as costimulatory blockade, which seeks to impede the activation of effector T cells [32]. Additionally, the administration of immunosuppressive drugs with precise targeting mechanisms is employed as part of the strategy. While immune tolerance induction approaches for TAV are currently in the experimental phase, preliminary studies have demonstrated the potential in mitigating or diminishing the severity of TAV in preclinical models and a limited number of clinical trials [31]. Additional research is required to enhance protocols and guarantee long-term safety and effectiveness.

In summary, immunotherapy exhibits significant promise for targeted treatment of cardiovascular conditions. The use of immune checkpoint inhibitors demonstrates potential in the reduction of plaque burden and stabilization of vulnerable plaques in cases of atherosclerosis. Cellular immunotherapies, such as mesenchymal stem cells (MSCs) and cardiac progenitor cells (CPCs), have demonstrated promising potential in facilitating cardiac tissue regeneration and enhancing post-myocardial infarction outcomes. Cytokine-based immunotherapies have the potential to modulate the immune response and facilitate cardiac repair in individuals with heart failure. The utilization of immune tolerance induction approaches, specifically focusing on regulatory T cells (Tregs), demonstrates potential in the prevention or treatment of transplant-associated vasculopathy. Although these applications are currently being investigated, they present promising opportunities for advancing the field of cardiovascular immunotherapy and enhancing patient outcomes.

Future directions and challenges in immunotherapy for CVD

Customized Immunotherapy for Cardiovascular Disease

Personalized immunotherapy holds significant promise as a prospective avenue for the advancement of cardiovascular disease (CVD) treatment. The objective is to customize therapeutic approaches for each patient by considering their distinct characteristics, such as immune profile, genetic background, and disease phenotype. By comprehending the distinct immune mechanisms that contribute to cardiovascular disease (CVD) in individual patients, the implementation of personalized immunotherapy has the potential to enhance treatment effectiveness while reducing the occurrence of adverse effects. The identification of molecular and immune signatures associated with various cardiovascular conditions has been made possible by recent advancements in high-throughput technologies, including next-generation sequencing and proteomics [33]. The utilization of these signatures can assist in the stratification of patients and provide guidance in the selection of suitable immunotherapeutic interventions. In the case of atherosclerosis, the ability to identify particular subsets of immune cells or cytokine profiles that are linked to plaque vulnerability could potentially facilitate the implementation of targeted immunomodulation strategies aimed at stabilizing vulnerable plaques [34].

In conjunction with immune profiling, genetic information plays a pivotal role in the development of personalized immunotherapy strategies. The presence of genetic variants in immune-related genes has the potential to impact both the response to treatment and the likelihood of experiencing adverse effects. Pharmacogenomic studies have the potential to identify genetic markers that can accurately predict the effectiveness and safety of immunotherapies [35]. This valuable information can then be used to make personalized treatment decisions for patients. In addition, recent developments in gene editing technologies, such as clustered regularly interspaced short palindromic repeats and CRISPR-associated protein 9 (CRISPR-Cas9), present the opportunity for accurate modification of immune cells or the targeting of specific genetic variants linked to cardiovascular disease (CVD) [34]. These approaches may allow for personalized immunotherapy strategies, including ex vivo gene editing of patient-derived immune cells for autologous cell therapies.

Utilization of Combination Therapies and Immunomodulatory Strategies

The future of cardiovascular disease (CVD) treatment shows great potential in the use of combination therapies that target multiple immune pathways or combine immunotherapy with conventional treatments. The intricate and multifaceted characteristics of cardiovascular disease (CVD) necessitate a comprehensive strategy that encompasses diverse facets of immune dysregulation and the development of cardiovascular pathology. The potential to enhance treatment efficacy exists by combining immune checkpoint inhibitors with other immunomodulatory agents, such as cytokines or targeted therapies. For instance, the combination of PD-1/PD-L1 blockade with agents that enhance regulatory T-cell function or suppress pro-inflammatory cytokines may potentially exert a synergistic effect on immune response modulation and contribute to the promotion of plaque stability in atherosclerosis [32]. In addition, the integration of immunotherapy with well-established cardiovascular treatments, such as lipid-lowering drugs or antiplatelet agents, has the potential to offer additional benefits. The implementation of combinatorial strategies that concurrently address conventional risk factors such as hyperlipidemia, while also modulating the immune response, may potentially yield a synergistic outcome in the prevention or treatment of cardiovascular disease (CVD) [36]. The investigation of combination therapies and immunomodulatory strategies necessitates a meticulous evaluation of treatment timing, dosing, and potential interactions among therapeutic agents. Preclinical and clinical studies play a crucial role in identifying optimal combinations and determining the most effective treatment regimens for various cardiovascular conditions.

Biomarkers for the Monitoring of Immunotherapeutic Response

The identification of dependable biomarkers is essential for monitoring the response to immunotherapy and informing treatment decisions in cardiovascular disease (CVD). Biomarkers play a crucial role in evaluating the effectiveness of treatments, forecasting clinical outcomes, and identifying patients who are most suitable for targeted immunotherapies [37]. Biomarkers can manifest in diverse forms, encompassing circulating immune cell subsets, cytokine profiles, genetic markers, or imaging modalities. For instance, the observation of alterations in distinct immune cell populations, such as regulatory T cells or pro-inflammatory T cells, could yield valuable information regarding the manipulation of the immune response and the efficacy of treatment [37]. In addition, molecular imaging techniques such as positron emission tomography (PET) or magnetic resonance imaging (MRI) can be utilized to evaluate plaque inflammation or metabolic activity [38]. Observations of alterations in plaque characteristics detected via imaging techniques have the potential to function as biomarkers for assessing treatment response and plaque stabilization. The validation and standardization of biomarkers are essential for ensuring their clinical utility. There is a need for extensive prospective studies to establish the reliability and predictive value of biomarkers in guiding immunotherapeutic interventions in cardiovascular disease (CVD). The integration of biomarker assessment into both clinical trials and routine clinical practice holds the potential to enhance personalized treatment decisions and optimize patient outcomes.

Safety Considerations and Potential Adverse Effects

The incorporation of safety considerations and the assessment of potential adverse effects are crucial elements to be addressed during the development and implementation of immunotherapy for cardiovascular disease (CVD). Although immunotherapies have demonstrated promising outcomes, they have the potential to elicit immune-related adverse events (irAEs) as a result of their systemic modulation of the immune system [39]. Idiopathic autoimmune diseases (irAEs) can exhibit a range of severity and have the potential to impact various organs, including the cardiovascular system. One example is myocarditis, which is characterized by inflammation of the heart muscle. It has been documented as a rare yet potentially life-threatening adverse event linked to immune checkpoint inhibitors [40]. It is crucial to comprehend the mechanisms that underlie immune-related adverse events (irAEs) and to identify risk factors or predictive markers. This understanding is vital in order to minimize adverse effects and enhance patient safety.

It is imperative to closely monitor patients who are undergoing immunotherapy in order to promptly identify and address any potential side effects. The implementation of strategies aimed at early detection and intervention is crucial for identifying cardiac-related adverse events. These strategies may involve the utilization of cardiac biomarkers or imaging techniques. The collaboration among clinicians, researchers, and regulatory bodies is crucial for ensuring ongoing safety evaluation, post-marketing surveillance, and the establishment of guidelines for effectively managing potential adverse effects related to immunotherapy in cardiovascular disease [41].

Regulatory and Logistical Challenges

One of the key issues that organizations face when operating in today's complex business environment is the regulatory and logistical challenges. These challenges can significantly impact the operations and success of a company, requiring careful consideration and strategy. The process of translating immunotherapy from laboratory research to clinical application encounters regulatory and logistical obstacles that must be successfully addressed in order to facilitate its widespread adoption [42]. Regulatory agencies play a pivotal role in the assessment of the safety and effectiveness of immunotherapies, ensuring that they adhere to stringent standards prior to granting approval [41]. Implementing measures to optimize regulatory processes and establishing well-defined guidelines can enhance the efficiency of the development and approval of immunotherapies for cardiovascular disease.

In addition, logistical challenges arise from the cost and accessibility of immunotherapies. Immunotherapies often incur significant costs and necessitate dedicated infrastructure and expertise for their administration, monitoring, and subsequent follow-up. It is imperative to allocate resources toward the optimization of manufacturing processes, cost reduction, and the establishment of implementation guidelines in clinical practice [43]. These measures are crucial in order to enhance the accessibility of immunotherapy to a wider range of patients. Effective collaboration among various stakeholders, such as researchers, clinicians, industry partners, regulatory agencies, and healthcare systems, plays a pivotal role in tackling regulatory and logistical challenges. Through collaborative efforts, these stakeholders can effectively support the advancement, assessment, and incorporation of immunotherapies into standard clinical procedures, ultimately enhancing the quality of patient care and outcomes in cardiovascular disease [44].

In summary, the future directions of immunotherapy in cardiovascular disease (CVD) encompass personalized approaches, combination therapies, treatment guided by biomarkers, optimization of safety measures, and the resolution of regulatory and logistical challenges. By making progress in these fields, we can leverage the potential of immunotherapy to transform the management of cardiovascular diseases and enhance patient outcomes.

## Conclusions

Immunotherapy presents a promising treatment modality for cardiovascular diseases (CVDs), providing innovative strategies to effectively target immunomodulatory mechanisms. The potential applications of immunotherapy in various cardiovascular conditions have been extensively discussed, emphasizing its significant therapeutic potential. However, there are challenges to address, including personalized treatment approaches, reliable biomarker identification, and ensuring safety in immunotherapeutic interventions. Additional research and clinical trials are required in order to comprehensively comprehend and effectively harness the potential of immunotherapy in the treatment of cardiovascular disease. Effective collaboration among researchers, clinicians, and regulatory bodies is of utmost importance in order to drive progress in the field. With continuous advancements, immunotherapy holds the potential to revolutionize the field of cardiovascular medicine. This could result in enhanced patient outcomes and a transformative impact on the management of cardiovascular disease.
